# Host-Plant Variations Affect the Biotic Potential, Survival, and Population Projection of *Myzus persicae* (Hemiptera: Aphididae)

**DOI:** 10.3390/insects12050375

**Published:** 2021-04-21

**Authors:** Muhammad Yasir Ali, Tayyaba Naseem, Muhammad Arshad, Ijaz Ashraf, Muhammad Rizwan, Muhammad Tahir, Misbah Rizwan, Samy Sayed, Muhammad Irfan Ullah, Rashad Rasool Khan, Muhammad Bilal Amir, Mingzhen Pan, Tong-Xian Liu

**Affiliations:** 1 College of Plant Health and Medicine, Qingdao Agricultural University, Qingdao 266109, China; m.yasirali4663@gmail.com (M.Y.A.); bilalamir6141@yahoo.com (M.B.A.); panmz2018@qau.edu.cn (M.P.); 2 Department of Botany, Government College Women University Faisalabad, Faisalabad 38000, Pakistan; tayyaba_nasem@yahoo.com; 3 Department of Entomology, University of Sargodha, Sargodha 40100, Pakistan; makuaf@gmail.com (M.A.); muhammad.irfanullah@uos.edu.pk (M.I.U.); 4 Institute of Agriculture Extension, Education and Rural Development, University of Agriculture, Faisalabad 38000, Pakistan; gilluaf707@uaf.edu.pk; 5 Rice Research Institute, Kala Shah Kaku, Sheikhupura 54000, Pakistan; misbahrizwanjabbar@gmail.com; 6 Department of Entomology, College of Agriculture and Environment, The Islamia University of Bahawalpur, Bahawalpur 63100, Pakistan; Tahir_1558@yahoo.com; 7 Department of Science and Technology, University College-Ranyah, Taif University, B.O. Box 11099, Taif 21944, Saudi Arabia; samy_mahmoud@hotmail.com; 8 Department of Entomology, University of Agriculture, Faisalabad 38000, Pakistan; rashadkhan@uaf.edu.pk

**Keywords:** *Myzus persicae*, age-stage two sex life table, insect-plant interaction, vegetables

## Abstract

**Simple Summary:**

Understanding insect–plant interactions is important in designing an integrated pest management program. Green peach aphid is a major insect pest of a wide range of crops. We studied the effects of five vegetable plants on the life table parameters of aphids. Our findings showed that cabbage was the less felicitous host plant to aphids for fecundity and survival. Contrarily, chili pepper was the most susceptible plant and was quite suitable for the faster growth and development of aphids. The information is very useful in designing the integrated pest management strategy against aphids.

**Abstract:**

The green peach aphid, *Myzus persicae* Sulzer (Hemiptera: Aphididae), a polyphagous insect pest is a major threat to a wide range of crops worldwide. Aiming to evaluate the life history traits of *M. persicae*, feeding on different host plants, we used five vegetables: cabbage, *Brassica oleracea* (Brassicaceae); chinese cabbage, *B. rapa* (Brassicaceae); chili pepper, *Capsicum annum* (Solanaceae); crown daisy, *Chrysanthemum coronarium* (Asteraceae); and eggplant, *Solanum melongena* (Solanaceae). TWOSEX-MSchart software was used for the statistical analysis about the age-stage, two-sex life table theory. The highest fecundity (69.65 individuals) rate of *M. persicae*, intrinsic rate of increase (*r* = 0.425 d^−1^), finite rate of increase (*λ* = 1.531 d^−1^), net reproductive rate (*R*_0_ = 69.65 offspring), and shortest mean generation time (T = 9.964 d) were recorded on the chili pepper plant. Whereas, lower fitness occurred on cabbage. The findings attained from population growth parameters indicate that chili pepper is the most susceptible plant, while cabbage is resistant to aphids. Population projection results also supported this statement, as the final total population size on cabbage was significantly lower than other plants. The reported information would be useful for devising integrated pest management programs, particularly those involving *M. persicae*. This information also suggests the adaptability of *M. persicae* causing economic damage to these vegetable cultivars.

## 1. Introduction

Phytophagous insects often feed on a wide range of plant species with different nutritional compositions, chemical defenses, and textures [1]. The adaptation of various host plants through selection pressure by insects’ feeding has led to the evolution of specific host-adapted populations [2]. The green peach aphid, *Myzus persicae* (Sulzer) (Hemiptera: Aphididae), is a polyphagous insect pest feeding on more than 400 species of plants from 40 different families and also vectors about 100 plant viruses [3,4]. Intensive infestation in the field lessens the plant vigor and cause serious damage due to loss of flower buds, weakness, water stress, and a general decline of vegetative growth [5,6,7]. *M. persicae* also feeds on vegetables and some ornamental plants, indicating its extensive genetic variation in accordance with host-plant adaptations. The higher adaptability of this pest allows better survival in diverse climatic conditions and on a variety of host plants [8].

Over the past few decades, several studies have evaluated the effectiveness of *M. persicae* on various host plant species [7,9,10]. The selection of genetically resistant or tolerant crops is one of the key components in integrated pest management programs [9,11]. The development of herbivorous insect pests may be directly related to the characteristics of host plants, such as the defensive compounds, nutritional value, and plant’s morphology including leaf pubescence, leaf toughness, presence or absence of trichomes, leaf shape, and color [12]. Defensive mechanisms in plants prolong the development of insect pests, leading to reduced fertility rates [13]. A higher fecundity rate and fast development of an insect pest indicates its fitness on that host plant [14]. Numerous studies have shown that plant species can affect the growth and reproduction of insect herbivores [11,15,16]. The study on insect–plant interaction is of utmost importance to attain high-yielding, high-quality, and aphid-resistant varieties of various plant species. The availability and quality of plant species directly affect the developmental period, fecundity, and survival, and the pest’s population growth rate; thus, a better understanding of these parameters on their host plants is crucial in developing an effective pest control program.

Life table theory is one of the most prevailing tools to compare the performance and fitness of pests on their host plant. It is very useful in implementing environment-friendly pest management programs as they reveal the combined effects of many biological parameters such as survival, fecundity, development, etc. on population growth [16,17]. Life table theory helps to measure the potential population growth of aphids, understanding the population dynamics, and estimation of reproductive growth parameters and insects’ potential [18,19]. This theory also provides the basic knowledge for various additional studies, such as mass rearing, behavioral analysis, and response to control agents, among others. Understanding the life table parameters of aphid on some important plants of the Solanaceae, Asteraceae, and Brassicaceae family can provide important information on developing an effective strategy for suppressing its population. Therefore, we examined the biological parameters of *M. persicae* on five different host plants according to age-stage, two-sex life table theory [20,21]. The findings of this study would be useful to better understand the survival, reproduction, development, and potential of aphid feeding on these important vegetables and could be helpful in designing future pest management programs for aphid species.

## 2. Materials and Methods

### 2.1. Host Plants

Five host plants—cabbage, *Brassica oleracea* (var. Jing feng yihao); chinese cabbage, *B. rapa* (var. Zaoshu nan you xiao baicai); crown daisy, *Chrysanthemum coronarium* (var. Xiao ye tong hao); eggplant, *Solanum melongena* (var. Guang jiazi hong chang qie); and chili pepper, *Capsicum annum* (var. Japanese Chao tianjiao)—were used in this study. The seeds were purchased from a local market and were sown in plastic pots. Plants were maintained throughout the experiment using a standard protocol. Twenty-seven plastic pots (10 cm diameter) were employed for each species of plant. The soil in pots consisted of peat moss and perlite substrate in a ratio of 3:1. The plants were kept in a growth chamber under the controlled conditions in an Intelligent Artificial Climate Box (Ningbo New Jiangnan Instrument Manufacturer Co., LTD., Zhejiang, China) at 25 ± 2 °C, 16:8 h light: dark photoperiod and 65–75% RH. Plants with fully developed 2–3 leaves were used to initiate the experiment.

### 2.2. Aphids

A colony of green peach aphid, *M. persicae* initially taken from the laboratory (Key Lab. of Insect Ecology and Molecular Biology of Qingdao Agriculture University, Shandong, China), was maintained on five corresponding host plants in a Climate Box having same conditions as described earlier.

### 2.3. Life Table Study

A group design was used to collect the life table data [11,22], having twenty-seven replicated pots per plant. The climatic chamber was set to provide a constant condition for experiment: temperature 25 ± 2 °C, relative humidity 65–75% and photoperiod 16:8 h (L:D). Ten apterous adult aphids were taken from the culture (described in Section 2.2) for each of five host plants and were placed individually on a leaf of their specific plant. Adult aphids were allowed to produce nymphs overnight and removed the day after. Only thirty new-born aphid nymphs were retained, and the surplus was removed. From the newly emerged nymphs, only one aphid was released on one plant to record life-history attributes. Twenty-seven aphids were used on twenty-seven plants for a single host species (totaling 27 × 5 = 135 aphids on 135 plants). Host plants with aphids were isolated within a plastic transparent cylindrical cage (10 cm in diameter, 30 cm high), which had the top and four-square sections (5 × 5 cm) cut out and covered in nylon mesh for ventilation; in addition, the leaf were enclosed in a nylon mesh cage on which the aphid was placed, somewhat similar with clip cage introduced by Haas et al. [23]. The number of aphids and their instars were recorded daily. Molting was determined by the presence of exuviae, a noticeable increase in body length, and by the presence of a cauda in case of adult females [24]. Data of four instars and adult were considered as single specimen [24]. Data for the life table attributes of *M. persicae* were recorded after every 24 h.

### 2.4. Population Projection

The projection of the population growth of *M. persicae* on the five host plants was calculated through Timing-MSChart [25], which is available at the same website for the TWOSEX-MSCHART program [26]. The age-stage two-sex life table data were used to project the population growth of aphids for next 60 days. The procedure for population growth rate projection was adapted as explained by Akca et al. [27]. For comparison of treatments, the initial population was set at 10 newborn nymphs in all treatments (host plants).

### 2.5. Statistical Analysis

The basic life table parameters such as age-stage survival rate (*s_xj_*), reproductive value (*v_xj_*), age-stage-specific life expectancy rate (*e_xj_*), intrinsic rate of increase (*r*), reproductive rate (*R*_0_), finite rate of increase (*λ*), and mean generation time (*T*) were analyzed using the computer program TWOSEX-MS Chart [26]. Based on the confidence interval of difference, the adult longevity, adult pre-oviposition period (APOP), total pre-oviposition period (TPOP), fecundity, and population parameters (*r*, *λ*, *R*_0_, and *T*) were compared using a quick paired bootstrapping technique. The bootstrapping technique in the program with 100,000 random samplings was used for calculating the SE for the population.

The age-specific survival rate (*l_x_*, *m_x_*_,_ and *R*_0_) was calculated as:

$${ɭ}_{x}=\sum_{j=1}^{k}S_{xj}$$

$$m_{x}=\frac{\sum_{j=1}^{k}S_{xj}f_{xj}}{\sum_{j=1}^{k}S_{xj}}$$

$$R_{o}=\overset{\infty }{\underset{x=0}{\sum }}l_{x}m_{x}$$

where *k* denotes the number of stages, *x* = age in days, *j* = stage, *R*_0_ (net reproductive rate) is the average number of offspring per female during its whole life cycle. It was calculated by the following equation.

The intrinsic rate of increase (*r*), finite rate of increase (*λ*), and mean generation time (*T*) is calculated as:

$$\overset{\infty }{\underset{x=0}{\sum }}e^{-r\left(x+l\right)}\;l_{x}m_{x}=1$$

$$\lambda =e^{r}$$

T=lnRo/r.

The life expectancy (E*_xj_*) is referred to the expected life of an individual of age *x* and stage *j* is calculated by following formula:

$$E_{xj}=\overset{\infty }{\underset{i=x}{\sum }}\cdot \overset{\beta }{\underset{y=j}{\sum }}{\text{ś}}_{iy}\;$$

where ś*_iy_* is the probability that individuals of age *x* and stage *j* will survive to age *i* and stage *y*, and it is calculated by considering ś = 1.

The reproductive value (*V_xj_*) was calculated by the equation suggested by Tuan et al. [28]:

$$V_{xj}=\frac{e^{r\left(x+1\right)}}{S_{xj}}\sum_{i=x}^{\infty }e^{-r\left(i+1\right)}\sum_{y=j}^{\beta }{\text{ś}}_{iy}f_{iy}.$$

## 3. Results

### 3.1. Age-Stage Two-Sex Life Tables

The data for the development time of each stage of *M. persicae* feeding on different hosts are given in Table 1. All nymphal instars (N1–N4) completed their development faster on eggplant and chili pepper. The pre-adult development period was highest when *M. persicae* was raised on cabbage (9.36 d), followed by crown daisy (6.19 d), chinese cabbage (5.96 d), chili pepper (5.62 d), and was shortest when raised on eggplant (5.48 d). Adult longevity was highest when *M. persicae* was fed on crown daisy (13.8 d), followed by cabbage (13.7 d), chili pepper (13.2 d), eggplant (12.8 d), and was shortest when fed on chinese cabbage (12.7 d). Total longevity was recorded highest on crown daisy (19.5 d) and shortest on cabbage (14.9 d). The *APRP* and *TPRP* was the period of the adult to first oviposition day and total time taken by an insect to first oviposition day, respectively. Highest *APRP* (0.36 d) and *TPRP* (9.71 d) were recorded on cabbage with a minimum lifelong fecundity (36.5 nymphs/female). Preadult survival was also recorded shorter on cabbage (0.5183). Highest lifelong fecundity was recorded on chili pepper (69.65 nymphs/female), followed by chinese cabbage (60.69 nymphs/female) (Table 1).

A highest net reproductive rate (*R*_0_) of *M. persicae* (69.6) was noted on chili pepper and the lowest (18.9) was recorded on cabbage. The highest to lowest values of intrinsic rate of increase were recorded on chili pepper (0.42), eggplant (0.42), chinese cabbage (0.41), crown daisy (0.34), and cabbage (0.21). The highest finite rates of increase (*λ*) were observed on chili pepper (1.53), eggplant (1.52), and chinese cabbage (1.51), while the lowest value of *λ* was recorded on cabbage (1.23). Mean generation time (*T*) was highest on cabbage (14.2 d) compared to other host plants (Table 2).

**Figure 1:**
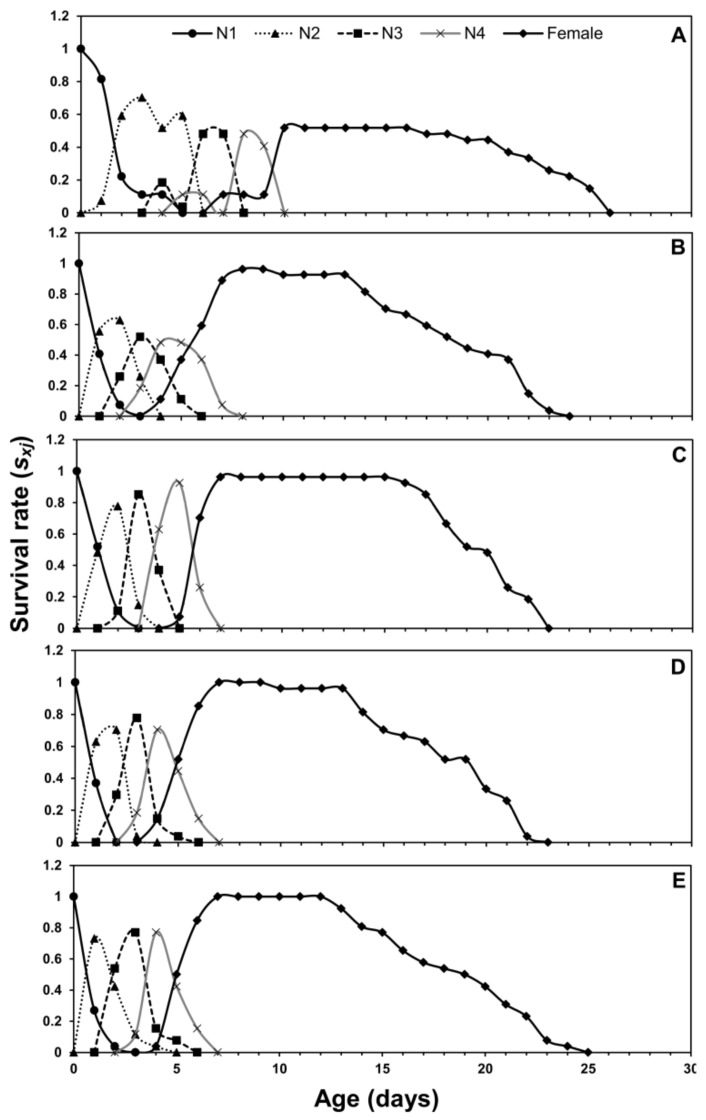
Age-stage-specific survival rate (*s_xj_*) of *Myzus persicae* fed on five host plants: cabbage (**A**), chinese cabbage (**B**), crown daisy (**C**), eggplant (**D**), and pepper (**E**).

**Figure 2:**
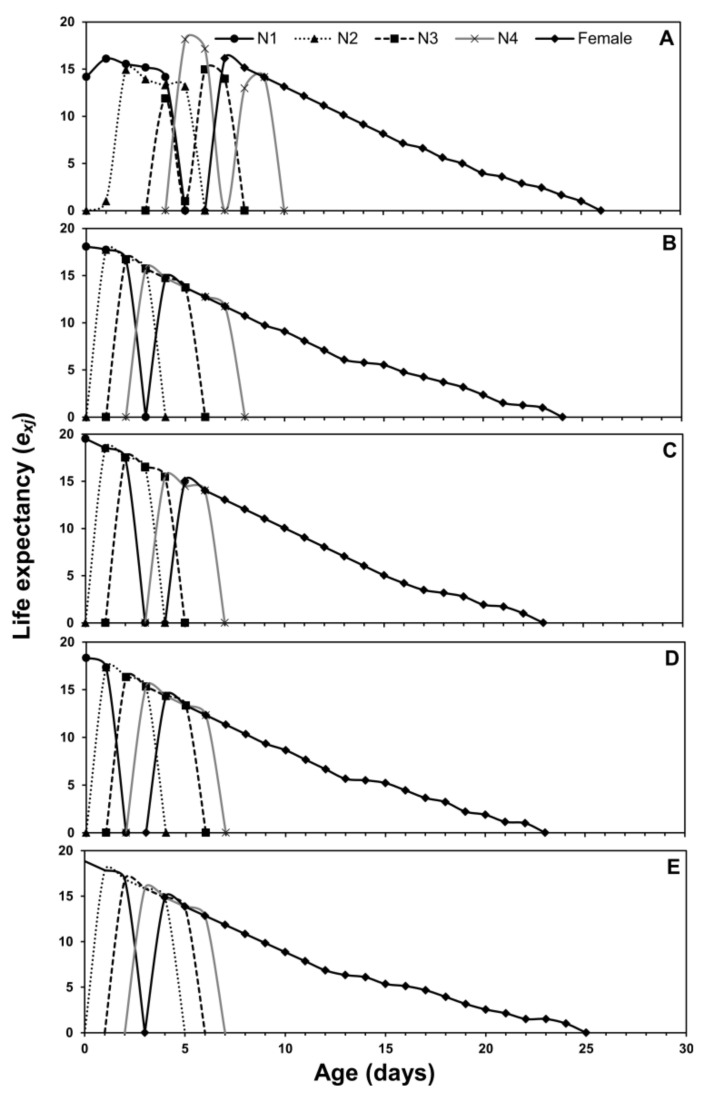
Age-stage-specific life expectance (*e_xj_*) of *Myzus persicae* fed on five host plants: cabbage (**A**), chinese cabbage (**B**), crown daisy (**C**), eggplant (**D**), and pepper (**E**).

**Figure 3:**
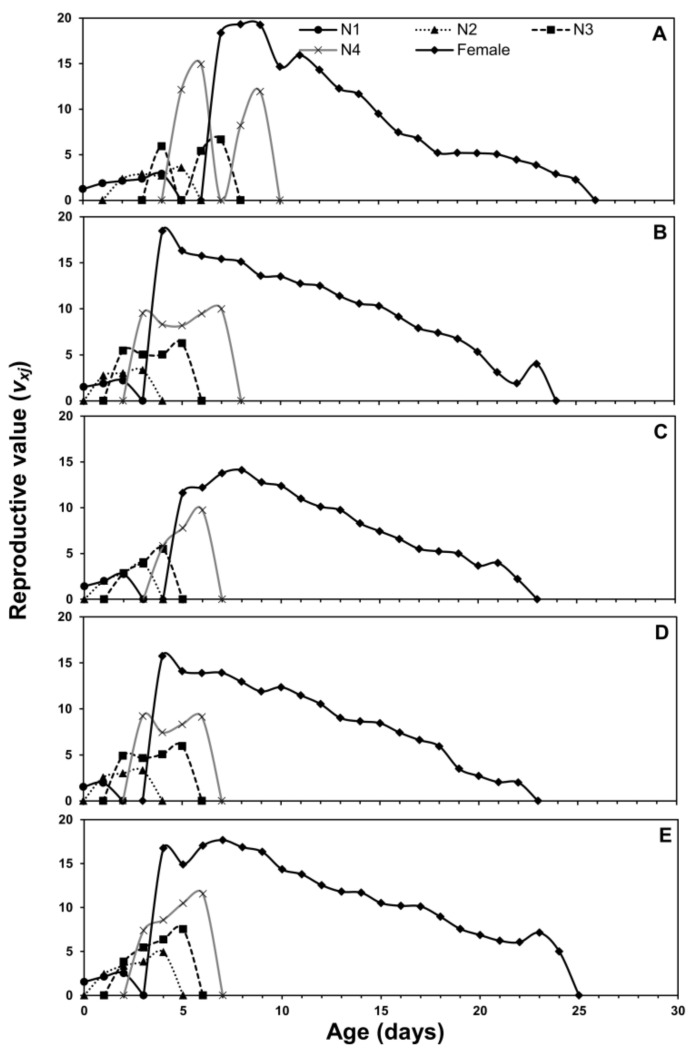
Age-stage-specific reproductive rate (*v_xj_*) of *Myzus persicae* fed on five host plants: cabbage (**A**), chinese cabbage (**B**), crown daisy (**C**), eggplant (**D**), and pepper (**E**).

**Figure 4:**
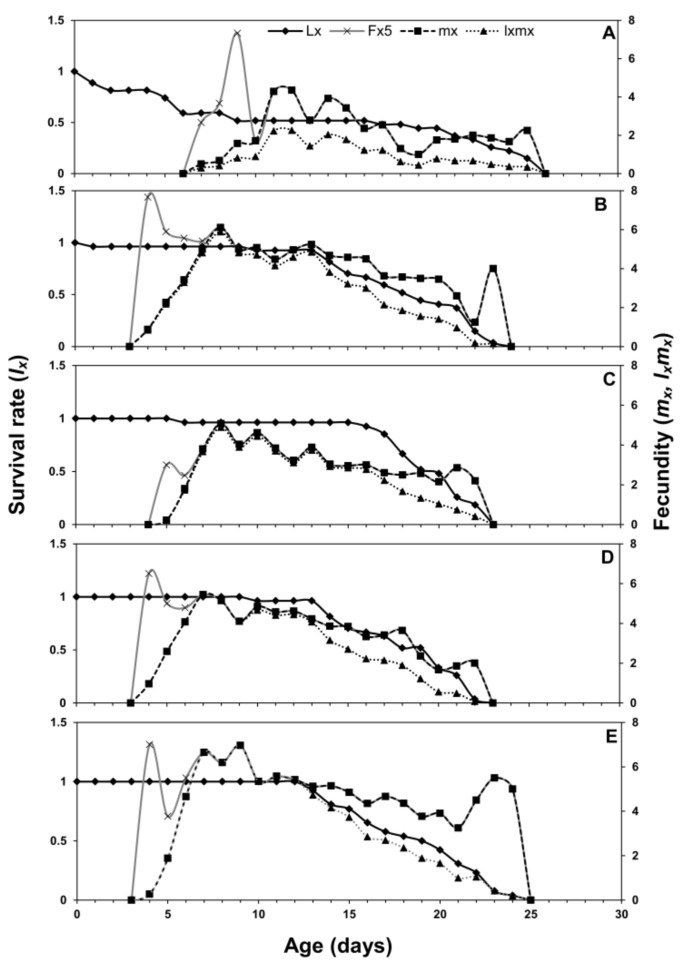
Age-specific survival rate (*l_x_*), age-stage-specific fecundity (*f_xj_*), age-specific fecundity (*m_x_*), and age-specific fertility (*l_x_m_x_*) of *Myzus persicae* fed on five host plants: cabbage (**A**), chinese cabbage (**B**), crown daisy (**C**), eggplant (**D**), and pepper (**E**).

Age-stage, two-sex life tables parameters describe the probability of a newborn to survive at a specific age (*x*) and stage (*j*). The age-stage curve (*s_xj_*) describes a higher survival rate on eggplant and chili pepper while lower on cabbage (Figure 1). The age-stage-specific life expectancy curve (*e_xj_*) is plotted in Figure 2. The newly born nymphs of *M. persicae* were expected to survive for a shorter period on cabbage when compared with other host plants. Females were expected to live a shorter life when fed on cabbage than other host plants. The age-stage-specific reproduction rate (*v_xj_*) for different host plants is plotted in Figure 3. Adult females contributed more to the population as they are the most productive stages of a population. The *l_x_*, *f_xj_*, and *m_x_* curves indicated that *M. persicae* had higher survival on chili pepper when compared with other host plants. The lowest fecundity was recorded when *M. persicae* fed on cabbage leaves (Figure 4).

### 3.2. Population Projection

**Figure 5:**
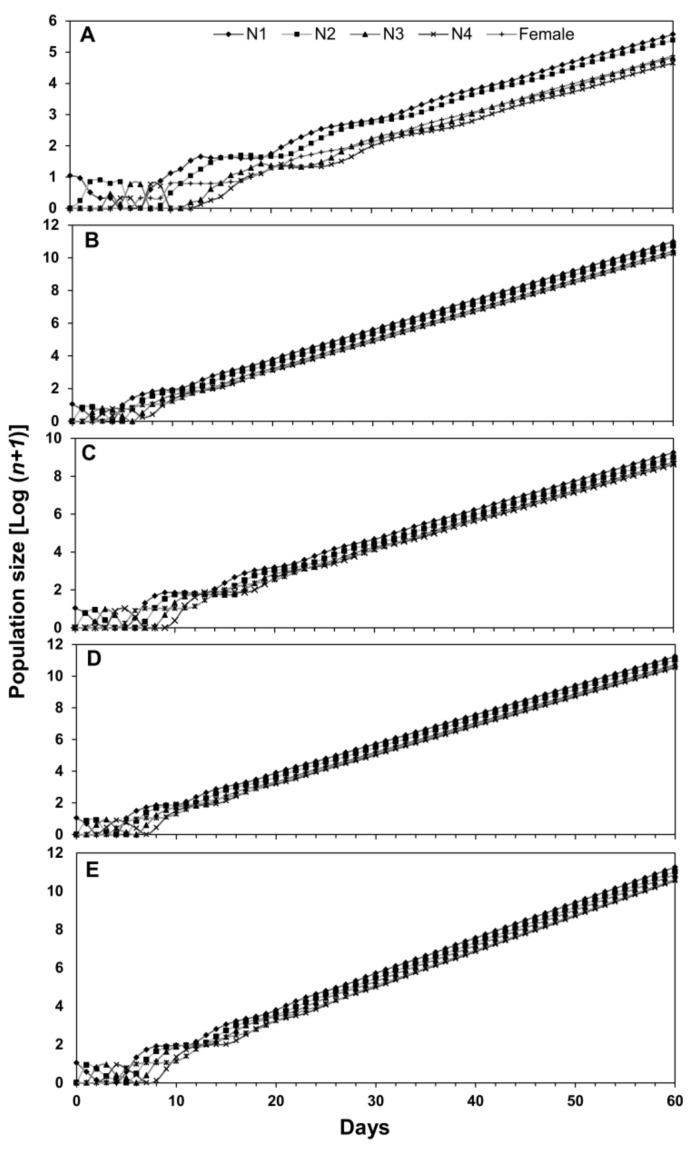
Population projection of *Myzus persicae* showing the changes in the age-stage structure fed on five host plants: cabbage (**A**), chinese cabbage (**B**), crown daisy (**C**), eggplant (**D**), and pepper (**E**).

The final total adult population sizes on cabbage, chinese cabbage, crown daisy, eggplant, and pepper host plats were 75,529.9; 19,920,246,234; 470,909,210.9; 42,349,041,261; and 41,583,141,580 individuals, respectively. The total population sizes of the *M. persicae* on the crown daisy, eggplant, and pepper were significantly larger than the populations observed on the cabbage (Figure 5).

## 4. Discussion

The availability and quality of host plants play a major role in the population dynamics of insect pests by affecting their developmental period, survival, and population parameters [7,29,30,31]. Many scientists have concluded that the performance of any aphid species can vary greatly on different host plants either on different cultivars or even varieties of the same plant [11,27,32]. To develop environmentally safe pest management strategies, complete knowledge of the population growth parameters of aphid on a wide variety of plant species is needed. Since these parameters provide a precise assessment of the growth rate of any insect pest population [28,33,34], they can be used to determine the tolerance level of any host plant to insect herbivores [9,35].

In this study, aphid performance was tested by feeding on five important vegetables under laboratory conditions. Cabbage was less felicitous to aphid than other plants. The lower intrinsic rate of increase and net reproductive rate on cabbage plant describe it as a poor host for *M. persicae* [11,16]. Our findings showed that the developmental period of small nymphal instars (N1 and N2) was longer when they fed on cabbage plant, while they grow faster on the chili pepper. The 3rd nymphal instars completed their development earlier on chinese cabbage as compared to cabbage and chili pepper. However, fully mature nymphs (N4) completed their developmental period faster on chili pepper as compared to cabbage. No significant difference was found in adult longevity when *M. persicae* fed on five different host plants. Similarly, the female fecundity was also higher when chili pepper and chinese cabbage plants were provided to *M. persicae* and the lowest fecundity rate was observed on cabbage. Female aphids showed no significant difference in oviposition (no. of days) when they fed on different host plants. Our findings showed that the population growth rate of aphids can be affected due to feeding on different host plants, and they refer to differences in the developmental period of immatures, female fecundity rate, and oviposition period. Many factors may affect the performance of aphids on their host plants, such as physicomorphic properties, nutritional value of the host plant, and its chemical composition [9,36]. Biological parameters such as the longevity, developmental period, survival, and fecundity rate of aphids depend on the physical and chemical properties of the host plant [37], as well as its nutritional value [38]. Further studies could be useful to identify specific factors in various vegetables that contribute to the growth of aphids.

We observed that *R*_0_, *r*, and *λ* of aphids were higher when they fed on chili pepper while their least values were recorded on cabbage plants. In our study, the highest intrinsic rate of increase (*r*) value for aphids was found in chili pepper treatment. The *r* is associated with the vulnerability of insect’s feeding [39]. In the demographic life table theory, if the value of *r* is greater than zero, the host is suitable for the insect’s population growth [40].

The *s_xj_* curves showed a higher survival rate of aphids on chinese cabbage and chili pepper, while the lowest survival rate is on cabbage. The *e_xj_* demonstrates that an adult is supposed to live at age *x* and stage *j* and it gradually reduces with age if there is no stress [41,42]. Values of *e_xj_* for aphids were higher when they fed on chili pepper as compared to other plants. The age-stage survival curves showed the preference of aphids on chili pepper plants as compared to other hosts. The age-stage, two-sex life table considers the variable developmental rate across the individuals, the overlapping curves of *s_xj_*, and the *l_x_* curve can simply describe the changes in the survival rates. Our findings support the previous work that described significant effects of different plant species on the growth, survival, and reproduction of insects [12,43]. The age-stage two-sex life table theory helps to construct a comprehensive life table describing the demographic characteristics of insect and mite populations. This tool allows the description of stage differentiation of *H. armigera* and incorporation of this factor into precise estimations of derived population parameters [20].

An accurate knowledge of a pest’s life table is important for executing an ecology-oriented management program. Further adding the consumption rate of each instar into life-table studies can be effectively characterized [44]. Thus, we suggest that the life table should be used in demographic studies of insects to get accurate population parameters for the population growth projections, to design a mass rearing program, to develop an effective pest management program, and to study the insect ecology. Our findings also illustrate that the projection populations can increase considerably faster when *M. persicae* fed on the crown daisy, chili pepper, and eggplant compared with the cabbage. These findings could be potentially useful to forecast the population growth of aphids on different host plants and devise an effective integrated management strategy for this pest. Further studies to identify the leaf morphology and phytochemicals such as plant volatiles and waxes in different host plants are suggested for a comprehensive discussion.

## 5. Conclusions

These results clarify the importance of using host–plant interactions in integrated pest control strategies to protect the crop from pest infestation. In addition, cabbage that was a less felicitous host for aphids can be considered as a potential source of resistance breeding programs. The use of less favorable host plants is economical for growers and has no adverse effect on other management strategies. The choice of a less susceptible host plant can be effective by reducing control costs and minimizing the negative effects of pesticide applications. In contrast, the most susceptible host plant (chili pepper) can be suitable for aphid-rearing in studies involving natural enemies of aphids.

## Figures and Tables

**Table 1:** Development period (Mean ± SE) of *M. persicae* raised on five different hosts.

Life Stage	*n*	Cabbage	*n*	Chinese Cabbage	*n*	Crown Daisy	*n*	Eggplant	*n*	Pepper
N1	54	2.42 ± 0.16 a	54	1.50 ± 0.09 b	54	1.63 ± 0.09 b	54	1.37 ± 0.07 c	52	1.31 ± 0.08 c
N2	38	3.26 ± 0.14 a	52	1.50 ± 0.07 b	54	1.41 ± 0.07 b	54	1.37 ± 0.07 b	52	1.31 ± 0.06 c
N3	32	1.81 ± 0.07 a	52	1.31 ± 0.06 b	54	1.33 ± 0.06 b	54	1.26 ± 0.07 b	52	1.54 ± 0.08 a
N4	28	2.00 ± 0.00 a	52	1.65 ± 0.09 b	52	1.85 ± 0.05 a	54	1.48 ± 0.07 c	52	1.46 ± 0.08 c
Preadult		9.36 ± 0.23 a		5.96 ± 0.16 bc		6.19 ± 0.07 b		5.48 ± 0.12 c		5.62 ± 0.11 c
Adult longevity	28	13.7 ± 0.41 a	52	12.7 ± 0.50 a	52	13.8 ± 0.28 a	54	12.8 ± 0.48 a	52	13.2 ± 0.52 a
Total Longevity		14.1 ± 1.33 b		18.1 ± 0.67 a		19.5 ± 0.46 a		18.3 ± 0.47 a		18.8 ± 0.518 a
Fecundity		36.5 ± 1.26 c		60.6 ± 2.68 a		47.1 ± 0.91 bc		54.3 ± 2.16 b		69.6 ± 2.42 a
TPRP		9.71 ± 0.29 a		6.00 ± 0.16 b		6.35 ± 0.09 b		5.52 ± 0.13 c		5.62 ± 0.11 c
APRP		0.36 ± 0.24 a		0.04 ± 0.03 b		0.15 ± 0.05 b		0.04 ± 0.03 b		0.00 ± 0.00 c
Ovi. days		12.1 ± 0.55 a		12.3 ± 0.52 a		13.6 ± 0.30 a		12.4 ± 0.05 a		12.9 ± 0.50 a
Preadult survival rate (*s_a_*)		0.51 ± 0.06 b		0.96 ± 0.02 a		0.96 ± 0.02 a		1.00 ± 0.00 a		1.00 ± 0.00 a

N1–N4 indicate the nymphal instar, means sharing similar letters in each row are not significantly different at *p* > 0.05, *n* = numbers of individual *M. persicae* that completed their development.

**Table 2:** Population parameters (Means ± SE) of *M. persicae* reared on five different hosts.

Parameters	Host Plants				
	Cabbage	Chinese Cabbage	Crown Daisy	Eggplant	Pepper
Net reproductive rate (*R*_0_) (offspring)	18.9 ± 2.57 d	58.4 ± 2.99 b	45.3 ± 1.48 c	54.3 ± 2.14 b,c	69.6 ± 2.38 a
Intrinsic rate of increase (*r*^−1^) (d^−1^)	0.21 ± 0.01 c	0.41 ± 0.01 a	0.34 ± 0.01 b	0.42 ± 0.01 a	0.42 ± 0.01 a
Finite rate of increase (*λ*) (d^−1^)	1.23 ± 0.01 c	1.51 ± 0.01 a	1.41 ± 0.06 b	1.52 ± 0.12 a	1.53 ± 0.01 a
Mean generation time (*T*) (d)	14.2 ± 0.40 a	9.83 ± 0.21 b	10.9 ± 0.12 b	9.43 ± 0.16 b	9.96 ± 0.17 b

Means sharing similar letters in each row are not significantly different at *p* > 0.05.

## Data Availability

The data presented in this study are available on request from the corresponding author.

## References

[1] Egan S.P., Ott J.R. (2007). Host plant quality and local adaptation determine the distribution of a gall-forming herbivore. Ecology.

[2] Drès M., Mallet J. (2002). Host races in plant-feeding insects and their importance in sympatric speciation. Phil. Trans. R. Soc. Lond. B.

[3] Blackman R.L., Eastop V.S. (2000). Aphids on the World’s Crops: An Identification and Information Guide.

[4] Meng J., Zhang C., Chen X., Cao Y., Shang S. (2014). Differential protein expression in the susceptible and resistant *Myzus persicae* (Sulzer) to imidacloprid. Pest Biochem. Physio..

[5] Herman M.A.B., Nault B.A., Smart C.D. (2008). Effects of plant growth-promoting rhizobacteria on bell pepper production and green peach aphid infestations in New York. Crop Prot..

[6] Saljoqi A.U.R., Khan K., Rahman S.U. (2009). Integrated management of potato-peach aphid, *Myzus persicae* (Sulzer). Sarhad J. Agric..

[7] La Rossa F.R., Vasicek A., López M.C. (2013). Effects of pepper (*Capsicum annuum*) cultivars on the biology and life table parameters of *Myzus persicae* (Sulz.) (Hemiptera: Aphididae). Neotrop. Entomol..

[8] Capinera J.L. (2011). Green Peach Aphid, Myzus persicae (Sulzer) (Insecta: Hemiptera: Aphididae).

[9] Goundoudaki S., Tsitsipis J.A., Margaritopoulos J.T., Zarpas K.D., Divanidis S. (2003). Performance of the tobacco aphid *Myzus persicae* (Hemiptera: Aphididae) on Oriental and Virginia tobacco varieties. Agric. For. Entomol..

[10] Mdellel L., Kamel M.B.H. (2014). Effects of different cultivars of pepper on the biological parameters of the green peach aphid. Eur. J. Environ. Sci..

[11] Polat Akköprü E., Atlıhan R., Okut H., Chi H. (2015). Demographic assessment of plant cultivar resistance to insect pests: A case study of the dusky-veined walnut aphid (Hemiptera: Callaphididae) on five walnut cultivars. J. Econ. Entomol..

[12] Awmack C.S., Leather S.R. (2002). Host plant quality and fecundity in herbivorous insects. Annu. Rev. Entomol..

[13] Bashir F., Azim N.M.N., Akhter N., Muzaffar G. (2013). Effect of texture/morphology of host plants on the biology of *Brevicoryne brassicae* (L.) (Homoptera: Aphididae). Int. J. Curr. Res..

[14] Liu J., Wu K., Hopper K.R., Zhao K. (2004). Population dynamics of *Aphis glycines* (Homoptera: Aphididae) and its natural enemies in soybean in northern China. Ann. Entomol. Soc. Am..

[15] Maremela M., Tiroesele B., Obopile M., Tshegofatso A.B. (2013). Effects of Brassica cultivar on population growth and life table parameters of the cabbage aphid, *Brevicoryne brassicae* L. (Hemiptera: Aphididae). J. Entomol. Res..

[16] Atlihan R., Kasap I., Özgökçe M.S., Polat-Akköprü E., Chi H. (2017). Population growth of *Dysaphis pyri* (Hemiptera: Aphididae) on different pear cultivars with discussion on curve fitting in life table studies. J. Econ. Entomol..

[17] Simon J.C., Peccoud J. (2018). Rapid evolution of aphid pests in agricultural environments. Curr. Opin. Insect Sci..

[18] Hosseini-Tabesh B., Sahragard A., Karimi-Malati A.A. (2015). laboratory and field condition comparison of life table parameters of *Aphis gossypii* Glover (Hemiptera: Aphididae). J. Plant Prot. Res..

[19] McDonald S.A., Halbert S.E., Tolin S.A., Nault B.A. (2003). Seasonal abundance and diversity of aphids (Homoptera: Aphididae) in a pepper production region in Jamaica. Environ. Entomol..

[20] Chi H., Liu H. (1985). Two new methods for the study of insect population ecology. Bull. Inst. Zool. Acad. Sin..

[21] Chi H. (1988). Life-table analysis incorporating both sexes and variable development rates among individuals. Environ. Entomol..

[22] Chang C., Huang C.Y., Dai S.M., Atlıhan R., Chi H. (2016). Genetically engineered ricin suppresses *Bactrocera dorsalis* (Diptera: Tephritidae) based on demographic analysis of group-reared life table. J. Econ. Entomol..

[23] Haas J., Lozano E.R., Poppy G.M. (2018). A simple, light clip-cage for experiments with aphids. Agric. Forest Entomol..

[24] Horsfall J.L. (1924). Life History Studies of Myzus persicae Sulzer.

[25] Chi H. (2020). TIMING-MSChart: A Computer Program for the Population Projection Based on Age-Stage, two-Sex Life Table.

[26] Chi H. (2020). TWOSEX-MSChart: A Computer Program for the Age-Stage, Two-Sex Life Table Analysis.

[27] Akca I., Ayvaz T., Yazici E., Smith C.L., Chi H. (2015). Demography and population projection of *Aphis fabae* (Hemiptera: Aphididae): With additional comments on life table research criteria. J. Econ. Entomol..

[28] Tuan S.J., Yeh C.C., Atlihan R., Chi H. (2016). Linking life table and predation rate for biological control: A comparative study of Eocanthecona furcellata (Hemiptera: Pentatomidae) fed on *Spodoptera litura* (Lepidoptera: Noctuidae) and *Plutella xylostella* (Lepidoptera: Plutellidae). J. Econ. Entomol..

[29] Davis J.A., Radcliffe E.B. (2008). Reproduction and feeding behavior of *Myzus persicae* on four cereals. J. Econ. Entomol..

[30] Razmjou J., Golizadeh A. (2010). Performance of corn leaf aphid, *Rhopalosiphum maidis* (Fitch) (Homoptera: Aphididae) on selected maize hybrids under laboratory conditions. Appl. Entomol. Zool..

[31] Obopile M., Ositile B. (2010). Life table and population parameters of cowpea aphid, *Aphis craccivora* Koch (Homoptera: Aphididae) on five cowpea *Vigna unguiculata* (L. Walp.) cultivars. J. Pest Sci..

[32] Madahi K., Sahragard A. (2012). Comparative life table of *Aphis pomi* (Hemiptera: Aphididae) on two host plants *Malus pumila* L. and *Chaenomeles japonica* under laboratory conditions. J. Crop Prot..

[33] Bussaman P., Sauth C., Chandrapatya A., Atlihan R., Gökçe A., Saska P., Chi H. (2017). Fast population growth in physogastry reproduction of *Luciaphorus perniciosus* (Acari: Pygmephoridae) at different temperatures. J. Econ. Entomol..

[34] Yu J.Z., Chen B.H., Güncan A., Atlihan R., Gökçe A., Smith C.L., Chi H. (2018). Demography and mass-rearing *Harmonia dimidiata* (Coleoptera: Coccinellidae) using *Aphis gossypii* (Hemiptera: Aphididae) and eggs of *Bactrocera dorsalis* (Diptera: Tephritidae). J. Econ. Entomol..

[35] Güncan A., Gümüs E. (2017). Influence of different hazelnut cultivars on some demographic characteristics of the Filbert aphid (Hemiptera: Aphididae). J. Econ. Entomol..

[36] Jahan F., Abbasipour H., Askarianzadeh A., Hassanshahi G., Saeedizadeh A. (2014). Biology and life table parameters of *Brevicoryne brassicae* (Hemiptera: Aphididae) on cauliflower cultivars. J. Insect Sci..

[37] Ulusoy M.R., Ölmez-Bayhan S. (2006). Effect of certain Brassica plants on biology of the cabbage aphid *Brevicoryne brassicae* under laboratory conditions. Phytoparasitica.

[38] Zarghami S., Allahyari H., Bagheri M.R., Saboori A. (2010). Effect of nitrogen fertilization on life table parameters and population growth of *Brevicoryne brassicae*. Bull. Insectol..

[39] Musa P.D., Ren S.X. (2005). Development and reproduction of *Bemisia tabaci* (Homoptera: Aleyrodidae) on three bean species. Insect Sci..

[40] Southwood T.R.E., Henderson P.A. (2009). Ecological Methods.

[41] Atlihan R., Chi H. (2008). Temperature-dependent development and demography of *Scymnus subvillosus* (Coleoptera: Coccinellidae) reared on *Hyalopterus pruni* (Homoptera: Aphididae). J. Econ. Entomol..

[42] Bailey R., Chang N.T., Lai P.Y. (2011). Two-sex life table and predation rate of *Cybocephalus flavocapitis* Smith (Coleoptera: Cybocephalidae) reared on *Aulacaspis yasumatsui* Takagi (Hemiptera: Diaspididae). Taiwan J. Asia-Pac. Entomol..

[43] Huang Y.B., Chi H. (2012). Age-stage, two-sex life table of *Bactrocera cucurbitae* (Coquillett) (Diptera: Tephritidae) with a discussion on the problem of applying female age-specific life table to insect populations. Insect Sci..

[44] Mitra S., Mobarak S.H., Barik A. (2001). Age-stage, two-sex life table of the biocontrol agent, *Altica cyanea* on three Ludwigia species. Biologia.

